# MSCI: an open-source Python package for information content assessment of peptide fragmentation spectra

**DOI:** 10.1093/bioadv/vbaf125

**Published:** 2025-05-24

**Authors:** Zahra Elhamraoui, Eva Borràs, Mathias Wilhelm, Eduard Sabidó

**Affiliations:** Proteomics Unit, Centre for Genomic Regulation (CRG), The Barcelona Institute of Science and Technology, Barcelona, 08003, Spain; Proteomics Unit, University Pompeu Fabra (UPF), Barcelona, 08003, Spain; Proteomics Unit, Centre for Genomic Regulation (CRG), The Barcelona Institute of Science and Technology, Barcelona, 08003, Spain; Proteomics Unit, University Pompeu Fabra (UPF), Barcelona, 08003, Spain; Computational Mass Spectrometry, Technical University of Munich, Freising, 85354, Germany; Munich Data Science Institute (MDSI), Technical University of Munich, Garching, 85748, Germany; Proteomics Unit, Centre for Genomic Regulation (CRG), The Barcelona Institute of Science and Technology, Barcelona, 08003, Spain; Proteomics Unit, University Pompeu Fabra (UPF), Barcelona, 08003, Spain

## Abstract

**Motivation:**

In mass spectrometry-based proteomics, the availability of peptide prior knowledge has improved our ability to assign fragmentation spectra to specific peptide sequences. However, some peptides exhibit similar analytical values and fragmentation patterns, which makes them nearly indistinguishable with current data analysis tools.

**Results:**

Here we developed the Mass Spectrometry Content Information (MSCI) Python package to tackle the challenges of peptide identification in mass spectrometry-based proteomics, particularly regarding indistinguishable peptides. MSCI provides a comprehensive toolset that streamlines the workflow from data import to spectral analysis, enabling researchers to effectively evaluate fragmentation similarity scores among peptide sequences and pinpoint indistinguishable peptide pairs in a given proteome.

**Availability and implementation:**

MSCI is implemented in Python and it is released under a permissive MIT license. The source code and the installers are available on GitHub at https://github.com/proteomicsunitcrg/MSCI.

## 1 Introduction

Mass spectrometry-based proteomics relies on the analysis of peptide fragmentation spectra for peptide identification and quantification (Eng *et al.* 1994, Aebersold and Mann 2003). The evaluation of fragmentation spectra has been achieved through several strategies, including the use of database search engines, *de novo* sequencing algorithms, or experimental spectral libraries (Noor *et al.* 2021). Machine-learning algorithms, such as MS2PIP (Degroeve and Martens 2013) and Prosit (Gessulat *et al.* 2019), have been used to predict several peptide properties, thereby increasing the available information to interpret fragmentation spectra and assign them to the correct peptide sequences. Despite the increased confidence in peptide identification provided by new rescoring algorithms like MS2Rescore and Oktoberfest (Declercq *et al.* 2022, Buur *et al.* 2024, Kalhor *et al.* 2024, Picciani *et al.* 2024), spectrum interpretation remains prone to ambiguities. This is especially noticeable when working in complex search spaces where peptides can exhibit similar fragmentation patterns and close retention times. An example of this scenario is illustrated by positional isomers of peptides with post-translational modifications such as glycosylation, acetylation, methylation, and phosphorylation, among others (Joyce and Searle 2024). The fact that these modifications can occur at different sites within the same peptide sequence results in peptides having identical m/z values, similar retention times, and closely related fragmentation patterns, thus complicating unequivocal spectra assignment. A similar situation arises in the fields of immunopeptidomics and metaproteomics, where the presence of similar peptide sequences and homologous proteins from distinct species introduces additional challenges (Nesvizhskii 2014, Blackburn and Martens 2016, Schiebenhoefer *et al.* 2019, Adams *et al.* 2020, Chong *et al.* 2022, Lichti *et al.* 2022). We have recently conducted a theoretical assessment to quantify the prevalence of indistinguishable peptides in certain search spaces (i.e. human canonical proteome and immunopeptidome) using state-of-the-art retention time and spectrum prediction models (Elhamraoui *et al.* 2024). Here, we present an open-source Python package designed to extend these assessments to other use cases, helping the users to identify those peptides that pose challenges to unequivocal identification within a given proteome of interest. The package allows users to analyze any set of proteins and proteomes, including post-translational modifications (PTMs) with available prediction models.

## 2 The MSCI Python package

The Mass Spectrometry Content Information (MSCI) Python package has been developed to facilitate, streamline, and expedite the identification of peptide sequences that pose challenges to unequivocal identification within a specific proteome or set of proteins. This includes peptide sequences with similar m/z values and retention times, which generate fragmentation patterns that are nearly indistinguishable from those of other sequences. The MSCI Python package relies on state-of-the-art proteomics mass spectrometry Python libraries such as matchms (Huber *et al.* 2021), Pyteomics (Goloborodko *et al.* 2013), and OpenMS (Röst *et al.* 2014, Pfeuffer *et al.* 2024), as well as, on extensively-tested general purpose computing packages like NumPy (Harris *et al.* 2020), Pandas (McKinney 2010), and Biopython (Cock *et al.* 2009).

The source code of MSCI can be freely accessed from GitHub under an MIT license (https://github.com/proteomicsunitcrg/MSCI) and the package is readily installable from PyPI using the standard pip module. The package is documented in https://msci.readthedocs.io/en/latest/ and a Google Colab Jupyter notebook was created to serve as a step-by-step learning tutorial for new users (https://colab.research.google.com/drive/1ny97RNgvnpD7ZrHW8TTRXWCAQvIcavkk). Finally, we also created a web-based version of MSCI that can be deployed locally or accessed in Streamlit-share (https://msci--proteomicsunit.streamlit.app/), which enables quick and easy data exploration without any further installation.

## 3 Overview of the MSCI workflow

**Figure 1.**
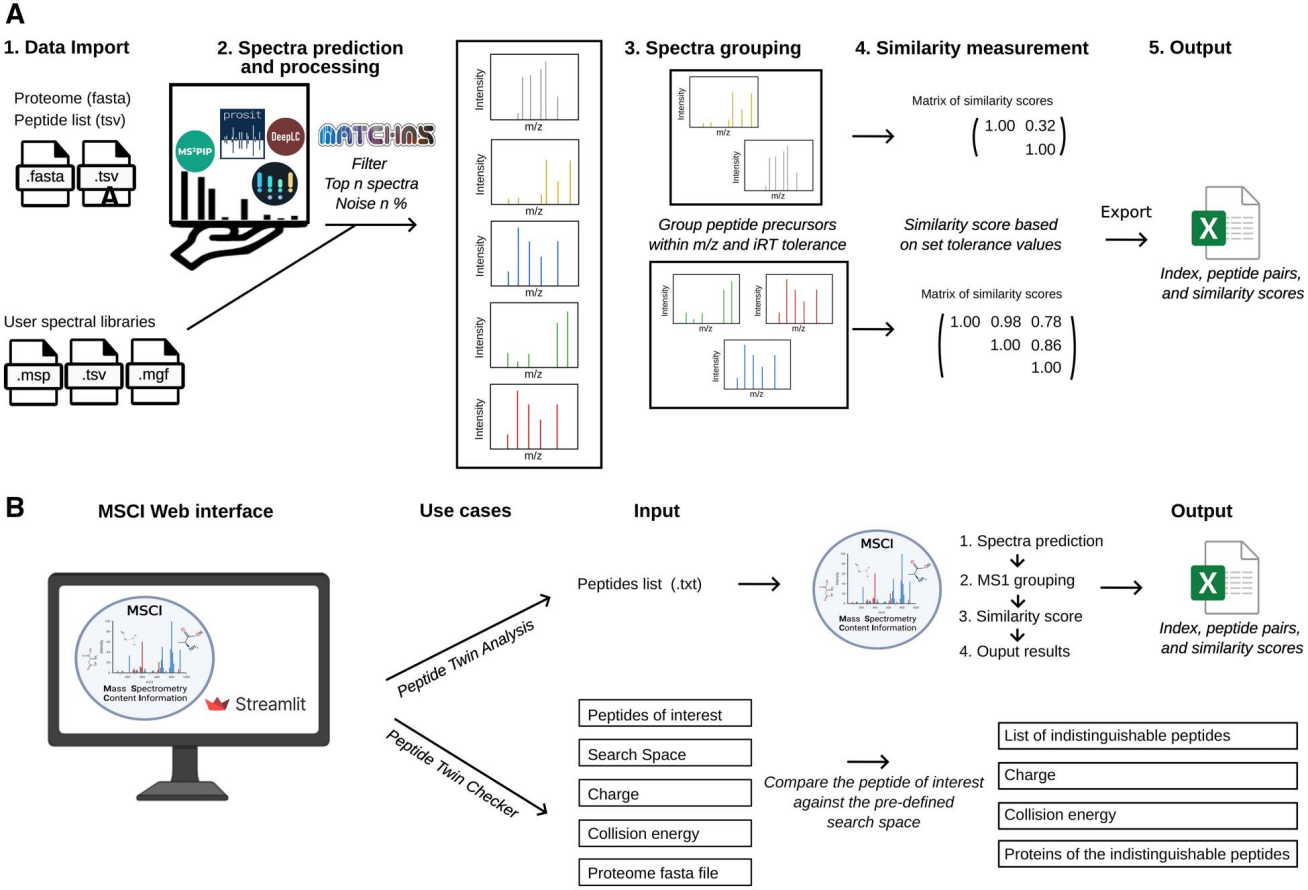
(A) Schematic representation of the different functionalities of the MSCI Python package, and (B) Overview of the web-based MSCI application workflow.

MSCI offers a variety of functionalities, including data parsing and spectral analysis, that are essential for calculating the similarity scores of peptide fragmentation spectra from a given set of proteins (see Fig. 1A). The key features of MSCI include:

### 3.1 Data import

MSCI can import either proteomes or spectral libraries. Entire proteomes can be imported as fasta files to subsequently perform *in-silico* digestion with different digestion rules (trypsin, Lys-C, chymotrypsin) or processed with sliding windows of variable lengths to support immunopeptidomics. Additionally, mutations can be introduced in the canonical protein sequences given a supporting file with Uniprot natural variants to enable proteogenomics applications. Proteomes can also be imported directly as peptide lists (*.tsv* format). In all cases, these peptide sequences will be later used to perform spectra prediction (see below). Alternatively, available experimental or in-silico spectral libraries can be directly imported in multiple file formats, including *.msp*, *.tsv*, and *.mgf*. The importing functions parse these files extracting the information related to the spectrum name, peptide m/z, charge, retention time if available, the fragment intensities and their m/z values. Extracted data are stored in Pandas’ data frames for easy manipulation and integration with other tools. The package provides methods to identify and remove duplicated peptide precursor entries.

### 3.2 Spectra prediction and processing

MSCI enables spectra prediction from *in-silico* digested proteomes or from peptide lists using Koina (Lautenbacher *et al.* 2024). The package allows users to customize the collision energy, the charge state, and the model for predicting fragmentation intensity and indexed retention time (iRT). Its integration with Koina enables MSCI users to utilize the already implemented input formats (LeDuc *et al.* 2018, 2022) and prediction models to meet specific research needs, and it ensures support for new formats and models as they become available—including properties like ion mobility, charge state, detectability, and post-translational modifications. The MSCI package includes spectra pre-processing tools to extract for the *n* most intense fragment ions or to filter out all fragment ions that do not reach a certain percentage of the maximum intensity.

### 3.3 Spectra grouping

The package provides functions to group peptide sequences based on MS1 m/z and iRT tolerances set by the user that are specified as Da or ppm for the m/z, and as dimensionless units for iRT. These functions allow for the effective grouping of peptides with similar m/z and iRT values, whose fragmentation spectra will be later compared to assess their similarity.

### 3.4 Similarity measurement

MSCI implements a Python adaptation of the peak matching functionality from the R package Spectra (Rainer *et al.* 2022). The joinPeaks class allows for flexible comparison of spectra based on user-defined m/z tolerance. The package includes functions to compute similarity measures between spectra, such as the normalized dot product, the normalized spectral angle, and the greedy cosine from matchms. Note that while the terms normalized dot product and cosine similarity are often used interchangeably, in this implementation the normalized dot product (*ndotproduct* function) incorporates additional weighting factors (*m* and *n*) for peak intensities during computation. To calculate the normalized spectral angle (*nspectraangle*) a weighted dot product of matched peak intensities is computed and the resulting value is then transformed into an angular measure and normalized to the range [0, 1]. The normalized spectral angle uses a peak matching process that bins peaks based on a set m/z tolerance and ppm thresholds. It iteratively matches peaks from two spectra using a heuristic approach that continues matching until no more candidates within tolerance remain. These scores are essential for identifying peptides with very similar fragmentation pattern, and thus assess spectral fragmentation uniqueness.

### 3.5 Output

As outputs, MSCI provides a table (in *.csv*, *.tsv*, or *.xlsx* format) listing peptide pairs and the similarity score resulting from comparing their fragmentation patterns. Moreover, the package also includes a plotting module to export the fragmentation spectra mirror plot (*.png* or *.svg* format) of those peptide pairs with a high similarity score.

Finally, we implemented a GUI for non-coders, in which the users are able to select functions and analyze their data directly in a web browser environment (Fig. 1B). The web version of the MSCI package offers two main use cases. The first is the “Peptide Twin Analysis,” which compares a list of peptides provided by the user applying different user-defined parameters (e.g., collision energy, charge, fragmentation prediction model, iRT prediction model, similarity score function, and m/z and iRT tolerances). MSCI calculates similarity scores among the different peptide pairs and outputs the list of all peptide pairs based on their m/z, iRT, and fragmentation pattern similarity scores. The second use case is the “Peptide Twin Checker,” where users can enter a peptide of interest and select a pre-calculated search space (e.g., human canonical proteome, human immunopeptidome). The MSCI website then checks whether the peptide sequence provided is indistinguishable from any other peptide within the selected search space.

## 4 Application of MSCI

To demonstrate the practical utility of MSCI, we conducted a theoretical assessment to quantify the prevalence of indistinguishable peptides within the human oral microbiome proteome using state-of-the-art retention time and spectrum prediction models. For this, we downloaded the Uniprot reference proteomes (251 reference proteomes, as in July 2024) listed in the oral human microbiome (https://www.homd.org) and processed as previously described (Elhamraoui *et al.* 2024). The reference UniProt database was initially submitted to *in-silico* strict tryptic digestion, allowing no miscleavages, and considering peptides with lengths 8–20 amino acids and charges +2 and +3. Based on these parameters, more than 10 million peptide precursors (i.e. 10 841 420 peptide ions) were obtained. For each peptide precursor, its fragmentation spectrum and iRT were predicted with Prosit (Prosit_2020_intensity_HCD) (Wilhelm *et al.* 2021) using high-energy collision-induced dissociation (HCD) at a normalised collision energy of 30 (NCE 30). Then, the predicted fragmentation spectra were compared for each peptide pair within 10 ppm m/z MS1 tolerance and ±5 iRT units (ca. 2.5 min in a 120-min chromatographic gradient), and their similarity was assessed using the normalised spectral angle function (10 ppm m/z MS2 tolerance). Based on our previous study, a normalized spectral angle threshold of 0.7 was set, indicating that peptide pairs with a similarity score above these thresholds were considered an indistinguishable pair. Based on our results, we observed 127 577 indistinguishable peptide precursors within the oral microbiome, which represents 1.18% of the total. We explored the concept of “peptide groups,” drawing an analogy to “protein groups.” We defined peptide groups as clusters of peptide precursors that are indistinguishable from one another based on their MS1 m/z, iRT, and fragmentation patterns. Upon applying this concept, we identified 61 672 distinct peptide groups. Most of these groups have two peptides but there are cases with more. An example of a peptide group containing several peptide precursors is the group ('IAAELGQR/2', 'LAAELANR/2', 'LAAELNAR/2', 'LAAENALR/2'), a case in which the peptide group consists of peptide precursor sequences with permutations and substitutions.

The analysis of the human oral microbiome builds on our previous efforts exploring the identification of indistinguishable peptide pairs and peptide groups in the canonical human proteome, the human proteome with natural variants, and the human immunopeptidome, which are also available through the “Peptide Twin Checker” use case on the web interface (Elhamraoui *et al.* 2024). Knowing which peptides are distinguishable from other sequences, and which are not, provides researchers with an important tool for defining the non-accessible portion of the proteome and allows for the prioritization of targets, such as immunopeptides and peptide biomarkers, that do not overlap ambiguously with other analytes.

## 5 Conclusion

The MSCI Python package was developed to tackle the challenges of peptide identification in mass spectrometry-based proteomics, particularly regarding indistinguishable peptides with similar precursor m/z, retention times, and fragmentation patterns. By integrating advanced computational tools, MSCI streamlines the workflow from data import to spectral analysis, enabling researchers to effectively assess fragmentation similarity scores among peptide sequences. This open-source tool enhances the ability to assess groups of peptide precursors that are indistinguishable in several search spaces and proteomics applications.

## Author contributions

Zahra Elhamraoui (Conceptualization, Data curation, Formal analysis, Software, Validation, Visualization, Writing—original draft), Eva Borràs (Conceptualization, Formal analysis, Investigation, Methodology, Validation, Writing—review & editing), Mathias Wilhelm (Conceptualization, Funding acquisition, Investigation, Methodology, Project administration, Resources, Software, Supervision, Validation, Writing—review & editing), and Eduard Sabidó (Conceptualization, Funding acquisition, Investigation, Methodology, Project administration, Resources, Software, Supervision, Validation, Visualization, Writing—review & editing)

## Conflict of interest

The authors declare the following financial interests/personal relationships which may be considered as potential competing interests: M. W. is a co-founder and shareholder of MSAID GmbH and OmicScouts GmbH, with no operational role in both companies.

## Data Availability

The source code and the installers are available on GitHub at https://github.com/proteomicsunitcrg/MSCI.
